# Ginsenoside RK3 Prevents Hypoxia-Reoxygenation Induced Apoptosis in H9c2 Cardiomyocytes via AKT and MAPK Pathway

**DOI:** 10.1155/2013/690190

**Published:** 2013-06-27

**Authors:** Jing Sun, Guibo Sun, Xiangbao Meng, Hongwei Wang, Min Wang, Meng Qin, Bo Ma, Yun Luo, Yingli Yu, Rongchang Chen, Qidi Ai, Xiaobo Sun

**Affiliations:** ^1^Key Laboratory of Bioactive Substances and Resources Utilization of Chinese Herbal Medicine, Ministry of Education, Institute of Medicinal Plant Development, Chinese Academy of Medical Sciences & Peking Union Medical College, Beijing 100193, China; ^2^Center for Translational Medicine and Jiangsu Key Laboratory of Molecular Medicine, Medical School of Nanjing University, Nanjing, Jiangsu 210093, China

## Abstract

Reperfusion therapy is widely utilized for acute myocardial infarction (AMI), but further injury induced by rapidly initiating reperfusion of the heart is often encountered in clinical practice. Ginsenoside RK3 (RK3) is reportedly present in the processed Radix notoginseng that is often used as a major ingredient of the compound preparation for ischemic heart diseases. This study aimed to investigate the possible protective effect of RK3 against hypoxia-reoxygenation (H/R) induced H9c2 cardiomyocytes damage and its underlying mechanisms. Our results showed that RK3 pretreatment caused increased cell viability and decreased levels of LDH leakage compared with the H/R group. Moreover, RK3 pretreatment inhibited cell apoptosis, as evidenced by decreased caspase-3 activity, TUNEL-positive cells, and Bax expression, as well as increased Bcl-2 level. Further mechanism investigation revealed that RK3 prevented H9c2 cardiomyocytes injury and apoptosis induced by H/R via AKT/Nrf-2/HO-1 and MAPK pathways. These observations indicate that RK3 has the potential to exert cardioprotective effects against H/R injury, which might be of great importance to clinical efficacy for AMI treatment.

## 1. Introduction

Acute myocardial infarction (AMI) is the most common cause of death and disability around the world. The pillar of current therapy for AMI is reperfusion to the affected area via thrombolytic therapy or angioplasty. However, reperfusion following ischemia/hypoxia induces further cardiomyocytes death, which is termed ischemia-reperfusion (I/R) injury [1]. Therefore, understanding the basis of reoxygenation and developing a cardioprotective drug that can alleviate the injury induced by I/R could maximize the benefits of reoxygenation therapy for AMI.

Although the underlying mechanism regulating myocardial injury induced by I/R is still not fully understood, apoptosis is shown to be a highly regulated program of cell death. Apoptosis is initiated shortly after the onset of myocardial infarction and becomes markedly enhanced during reperfusion [2, 3]. Thus, restraining the cardiomyocyte apoptosis induced by I/R can result in improved prognosis of AMI.

The phosphatidylinositol 3-kinase (PI3K)/AKT and mitogen-activated protein kinases (MAPKs) signaling pathways are known to play pivotal roles in controlling the survival and apoptosis of cardiomyocytes [4–6]. Various studies demonstrated that AKT phosphorylation can activate nuclear factor-erythroid 2- related factor 2 (Nrf-2), which controls the expression of various antioxidant enzymes and Phase II detoxification enzymes such as heme oxygenase-1 (HO-1). HO-1, a subtype of heme oxygenase (HO), plays a central role in cellular antioxidant defense. Many reports have indicated that upregulation of HO-1 mediated by AKT phosphorylation plays an important role in promoting cell survival and protecting against H/R injury in cardiomyocytes [7–9]. Much evidence shows that MAPKs, which include c-Jun NH_2_-terminal kinases (JNKs), extracellular signal-regulated protein kinase (ERK1/2), and p38 kinases, play critical roles in cells survival and apoptosis during IR injury [10]. The role of the ERK1/2, JNK, and P38 pathway in apoptosis remains controversial, as both proapoptotic and antiapoptotic effects have been observed dependenting on cell type and apoptotic stimuli [11–14]. 

Radix notoginseng are frequently used in the prevention and treatment of cardiovascular diseases in China and other Asian countries. Panax notoginseng saponins (PNS), including notoginsenoside R1, ginsenosides Rg1, Rb1, Rh2, and RK3, are generally believed to be the main active components responsible for the claimed efficacy [15–18]. Ginsenoside RK3 (RK3) is reportedly present in the processed Radix notoginseng herbs [19]. Previous experiments showed that RK3 possesses immunomodulatory, antiplatelet aggregating, and antiproliferative activity [20–22]. However, little is known about the possible cardioprotective effect of RK3. Therefore, exploring the potential cardioprotective effect of RK3 and its underlying mechanisms is of great interest.

## 2. Materials and Methods 

### 2.1. Materials

**Figure 1:**
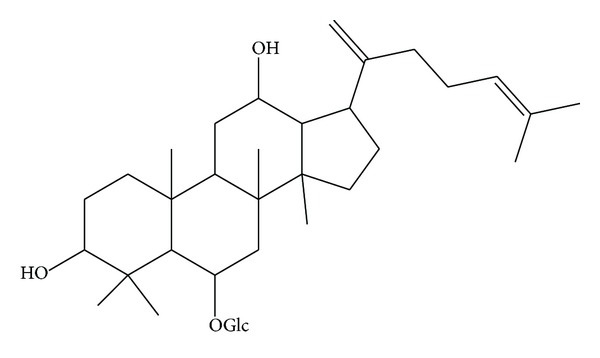
Molecular structure of Ginsenoside RK3.

Ginsenoside RK3 (molecular weight = 620; purity > 98%) was purchased from Shanghai Winherb Medical S&T Development (Shanghai, China). The molecular structure of RK3 is shown in Figure 1. Rat embryonic cardiomyoblast-derived H9c2 cardiomyocytes were obtained from the Cell Bank of the Chinese Academy of Sciences (Shanghai, China). All cell culture materials were from GIBCO (Grand Island, NY). The Cell Counting Kit-8 was purchased from Dojindo laboratory (Japan). Caspase-3 fluorometric and ROS fluorometric assay kits were acquired from BioVision (CA, USA). The kits for determining lactate dehydrogenase (LDH) were purchased from Nanjing Jiancheng Institute of Biological Engineering (Nanjing, China). All antibodies were purchased from Santa Cruz Biotechnology (Santa Cruz, CA), and other chemicals were purchased from Sigma (St. Louis, MO). 

### 2.2. Cell Culture and Hypoxia-Reoxygenation

H9c2 cardiomyocytes were cultured in high glucose DMEM supplemented with 10% (v/v) fetal bovine serum, 1% penicillin/streptomycin (v/v), and 2 mM L-glutamine. The cells were maintained at 37°C with 100% relative humidity in a CO_2_ incubator containing 5% CO_2_ at 37°C. High glucose DMEM medium was changed with none glucose DMEM to mimic ischemia. Then the H9c2 cardiomyocytes were incubated at 37°C in an anaerobic glove box (Coy Laboratory, USA), from which normal air was removed by a vacuum pump and replaced with 5% CO_2_, 5% H_2_, and 90% N_2_. The H9c2 cardiomyocytes were cultured under hypoxia for 6 h. Then the cells were removed from the anaerobic glove box, and the medium was replaced with high glucose medium and maintained in the regular incubator to mimic reperfusion. The corresponding control cells were incubated under normoxic conditions for equivalent durations with high glucose DMEM. For all experiments, cells were plated at an appropriate density according to the experimental design and were grown for 24 h to reach 70%–80% confluence before experimentation.

### 2.3. Analysis of Cytotoxicity

Cytotoxicity was determined by two alternative methods: gross detection of cell viability by Cell Counting Kit-8 (CCK8) assay and cell death by LDH assay. In cell viability assay, H9c2 cardiomyocytes were seeded at 1 × 10^4^ cells/well in 96-well plates. After 6 h of hypoxia treatment, cell viability was determined at 0, 2, 4, 8, 12, and 24 h after reoxygenation according to the CCK8 assay kit protocol. Briefly, 10 *μ*L of CCK8 solution was added to the culture medium. Optical density was measured at 450 nm wavelength and afterwards incubated for an additional 2 h, using a microplate reader (SpectraFluor, TECAN, Sunrise, Austria). Next, prior to H/R treatment, the cells were incubated with different concentrations of RK3 (6.25, 12.5, 25, and 50 *μ*g/mL) for 12 h, and then cell viability was evaluated as mentioned earlier. 

Cell death was evaluated by LDH method. Briefly, H9c2 cardiomyocytes were cultured at 2 × 10^5^ cells/well in 6-well plates for 24 h. After H/R treatment with or without RK3 (25 *μ*g/mL) pretreatment, the medium was collected to measure LDH release using LDH assay kits, following manufacturer instructions. 

### 2.4. Analysis of Caspase-3 Activation

The activation of caspase-3 was determined using a fluorescein active caspase-3 staining kit (BioVision), following the instructions supplied by the manufacturer. Briefly, H9c2 cardiomyocytes were collected after H/R treatment with or without RK3 (25 *μ*g/mL) pretreatment and then incubated on ice with 50 *μ*L chilled lysate buffer for 10 min. Next, 50 *μ*L of 2x reaction buffer (containing 10 mM dithiothreitol) and 5 *μ*L of caspase-3 substrate (DEVD-AFC, 1 *μ*M) were added to each sample. The specimens were then incubated at 37°C for 2 h. Caspase-3 activity was determined by measuring fluorescence at an excitation wavelength of 380 nm and an emission wavelength of 440 nm (SpectraFluor, TECAN, Sunrise, Austria).

### 2.5. Hoechst 33342 and PI Double Staining

In this study, cells were double-stained with Hoechst 33342 and propidium iodide (PI) for the qualitative analysis of apoptosis. H9c2 cardiomyocytes were cultured in 24-well plates for 24 h. After treatment, cells were washed twice with phosphate-buffered saline (PBS) and incubated with 10 *μ*g/mL Hoechst 33342 dye for 15 min at 37°C in the dark, and then 100 *μ*g/mL propidium iodide was added (PI, Sigma). Stained nuclei were observed immediately, using a fluorescence microscopy (Leica, Germany Q9). In normal cells, the nuclei appeared intact and even stained in blue by Hoechst 33342, whereas cells with bright blue or red/pink nuclei were considered apoptotic cells [23]. 

### 2.6. Terminal Deoxynucleotidyl Transferase-Mediated dUTP Nick End Labeling (TUNEL) Staining

Terminal-deoxynucleotidyl-transferase- (TdT-) mediated desoxyuridinetriphosphate (dUTP) nick end labelling (TUNEL) was used in apoptosis detection. Briefly, H9c2 cardiomyocytes were cultured in 24-well plates for 24 h. After exposure to hypoxia for 6 h and reoxygenation for 24 h, H9c2 cardiomyocytes were fixed by incubation in 10% neutral buffered formalin solution for 30 min at room temperature. H9c2 cardiomyocytes were incubated for 30 min with a methanol solution containing 0.3% H_2_O_2_ to stop the activity of endogenous peroxidase. H9c2 cardiomyocytes were treated with a permeabilizing solution (0.1% sodium citrate and 0.1% Triton X-100) for 2 min at 4°C and then incubated in the TUNEL reaction mixture for 60 min at 37°C. Morphological analysis was performed through fluorescence microscopy (DM4000B, Leica, Wetzlar, Germany). Four fields were randomly selected from each sample, and at least 100 cells were counted to calculate the apoptosis rate.

### 2.7. Preparation of Cell Lysates for Western Blotting

H9c2 cardiomyocytes were pretreated with RK3 (25 *μ*g/mL) for 12 h. The cells were harvested after H/R, washed once with PBS, and then the cytoplasmic and nuclear fractions were lysed by commercially available cytoplasmic extraction reagents on ice. The supernatants were collected, assayed for protein concentration using bicinchoninic acid assay, and stored at −80°C until their use in Western blot analysis. 

### 2.8. Western Blot Analysis

Equal amounts (10 *μ*g) of protein fractions were separated by electrophoresis on 10% sodium dodecyl sulfate polyacrylamide gels (SDS-PAGE), in which the protein samples were evenly loaded. The proteins were then transferred onto nitrocellulose membranes in Tris-glycine buffer at 110 V for 1 h. The membranes were blocked with 5% (w/v) nonfat milk powder in Tris-buffered saline containing 0.1% (v/v) Tween-20 (TBST) and then incubated overnight with appropriate primary antibodies at 4°C. Afterwards, they were washed thrice with TBST and incubated with secondary antibodies for 2 h at room temperature. The results were visualized by enhanced chemiluminescence.

### 2.9. Statistical Analyses

The results were expressed as means ± standard deviation. All statistical analyses were performed through Student *t*-test or analysis of variance (ANOVA) with Prism 5.00 software. Statistical significance was considered at *P* < 0.05. All data were performed in at least three independent experiments.

## 3. Results

### 3.1. Effect of H/R on Cell Viability and Cell Death in H9c2 Cardiomyocytes

**Figure 2:**
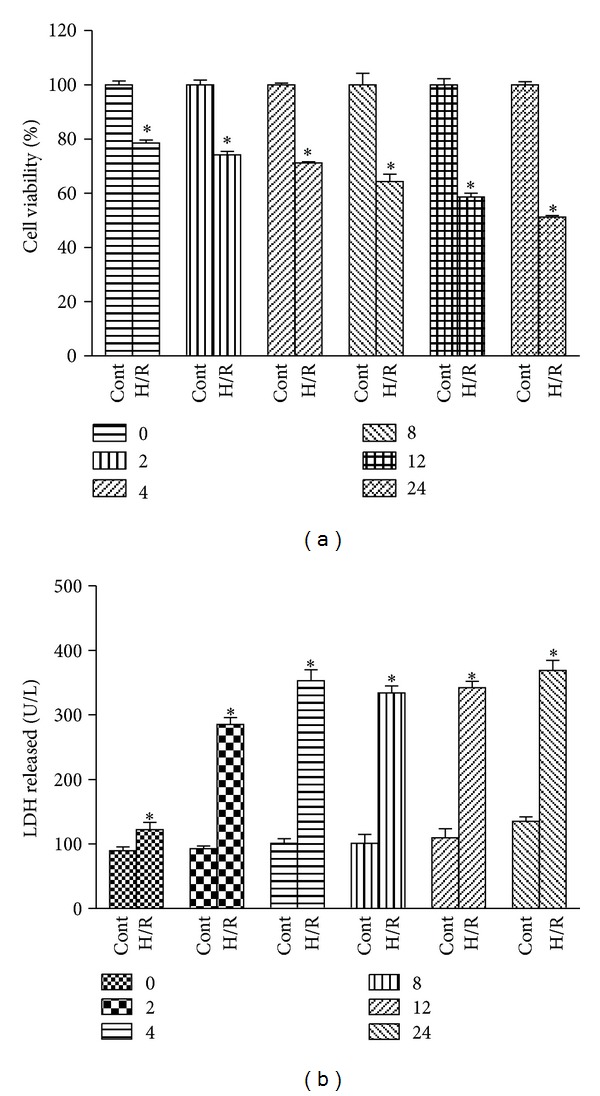
Effects of H/R on cell viability and LDH release in H9c2 cardiomyocytes. H9c2 cardiomyocytes were exposed to 6 h of hypoxia, followed by 24 h of reoxygenation. Then, cell viability was detected at 0, 2, 4, 8, 12, and 24 h after reoxygenation using CCK8 assay (a). Cell death was also measured by LDH assay kit (b). The results are represented as means ± SD from three independent experiments. *P < 0.05 versus control group.

In order to study whether RK3 was able to protect against cardiac injury induced by H/R in vitro, we first determined the reoxygenation conditions leading to cell toxity in H9c2 cardiomyocytes. Cells were exposed to hypoxia for 6 h, followed by reoxygenation for 24 h. Then, cell viability was detected at 0, 2, 4, 8, 12 and 24 h after reoxygenation by CCK8 assay. The cells in control group were considered 100% viable. As shown in Figure 2(a), 6 h of hypoxia caused a decrease of approximately 25.46% in cell viability, and reoxygenation provoked further decline in cell viability by a time-dependent manner. The viability of cardiomyocytes at 24 h after reoxygenation was around 51.17%. 

LDH leakage, as a biomarker of cell death, was also detected at different times of reoxygenation. As shown in Figure 2(b), reoxygenation induced further release of LDH compared to hypoxia groups. LDH leakage increased quickly at 0 h to 4 h after reoxygenation and reached a peak at 24 h. Based on the results previous, hypoxia for 6 h and reoxygenation for 24 h were selected as optimal conditions for the following experiments.

### 3.2. Effect of H/R on Cell Apoptosis in H9c2 Cardiomyocytes

**Figure 3:**
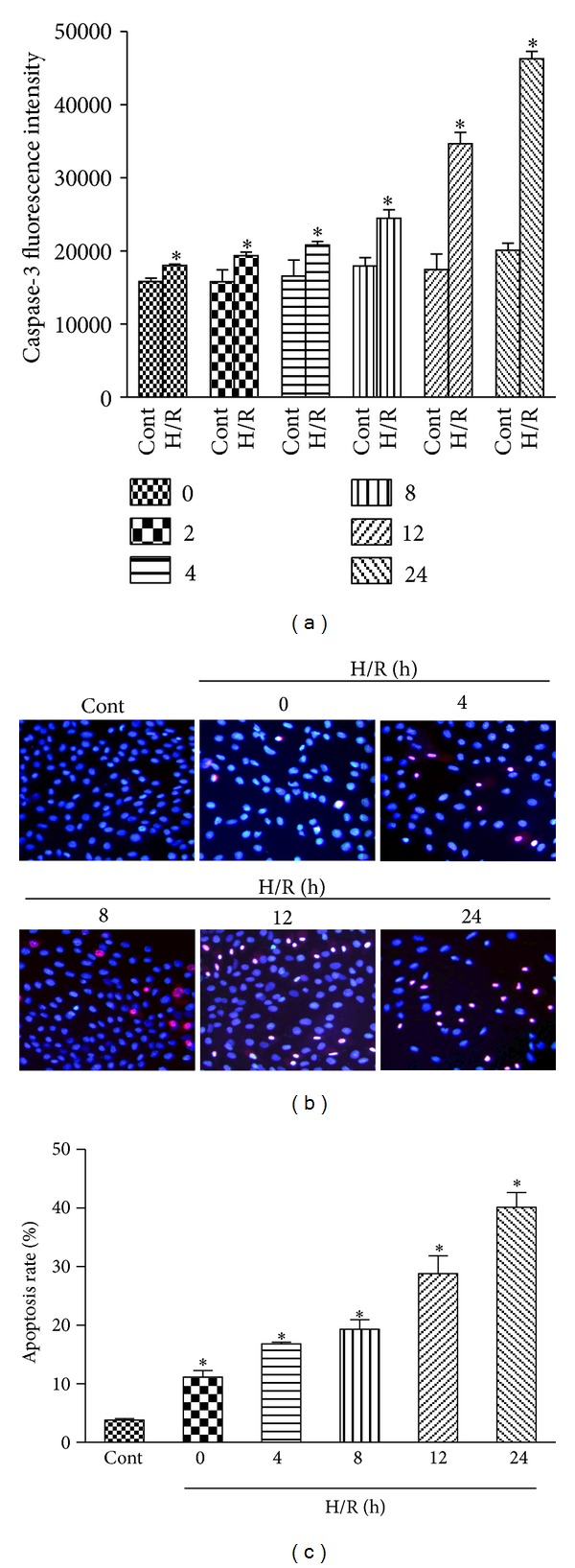
Effects of H/R on apoptosis in H9c2 cardiomyocytes. H9c2 cardiomyocytes were exposed to 6 h of hypoxia followed by 24 h of reoxygenation. Then, the caspase-3 activity (units per microgram of protein) was assayed, as described in Section 2, at 0, 2, 4, 8, 12, and 24 h after reoxygenation (a). Hoechst 33342 and PI double staining were also used in the qualitative and quantitative analyses of the apoptotic cells (b and c). The results are represented as means ± SD from three independent experiments. *P < 0.05 versus control group.

H9c2 cardiomyocytes were exposed to hypoxia for 6 h, and caspase-3 activity, a biomarker of apoptosis, was detected at different times after reoxygenation to assess whether this cytotoxic effect was connected with apoptosis to some extent. As shown in Figure 3(a), the reoxygenation induced activation of caspase-3, which started at 2 h after reoxygenation and reached an activation peak at 24 h. This result indicated that cardiac myocyte apoptosis contributed to myocardial H/R injury.

The morphological changes in apoptotic H9c2 cardiomyocytes induced by H/R were observed through Hoechst 33342/PI staining. Cells with blue nuclei were considered normal, while cells with bright blue or red/pink nuclei were considered apoptotiosis. As shown in Figure 3(b), few cells with nuclear staining bright blue or red/pink were observed in the control group. After being exposed to hypoxia for 6 h, about 11.16% of cells showed apoptosis characteristics. Reoxygenation significantly increased the percentage of the apoptotic cells in a time-dependent manner compared with the groups that accepted hypoxia alone, as shown in Figure 3(c).

### 3.3. Effect of H/R on AKT and MAPK Signaling Pathways in H9c2 Cardiomyocytes

**Figure 4:**
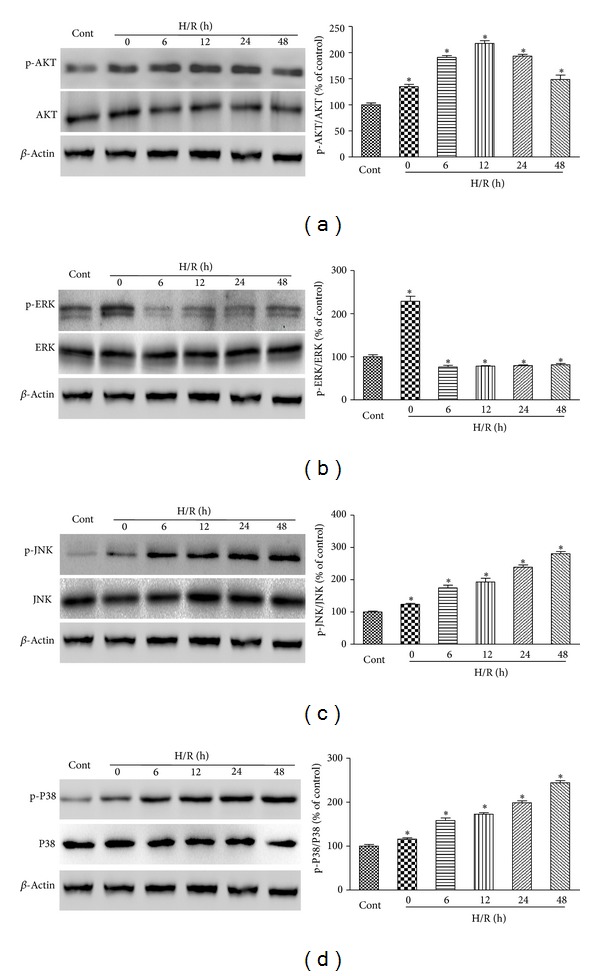
Effects of H/R on AKT and MAPK signaling pathways in H9c2 cardiomyocytes. H9c2 cardiomyocytes were exposed to 6 h of hypoxia followed by 48 h reoxygenation. Then the expression levels of phosphorylated and total AKT (a), ERK1/2 (b), JNK (c), and p38 (d) were detected by immunoblotting assay at 0, 6, 12, 24, and 48 h after reoxygenation. The percentage values of the p-AKT/AKT, p-ERK1/2/ERK1/2, p-JNK/JNK, and p-p38/p38 ratios relative to the control condition are shown. Normalization of Western blots was ensured by *β*-actin. The results are represented as means ± SD from three independent experiments. *P < 0.05 versus control group.

We examined the protein expression of total and phosphorylated (active form) AKT and three major constituents of the MAPK signaling cascade, ERK1/2, JNK, and p38 at different times (0 h to 48 h) to elucidate the molecular mechanism of H/R-induced apoptosis in H9c2 cardiomyocytes. As shown in Figure 4, phosphorylation of AKT, ERK1/2, JNK, and p38 was markedly induced after hypoxia. Increased p-AKT, p-JNK, and p-p38 remained active until 48 h after reoxygenation (Figures 4(a), 4(c), and 4(d)). By contrast, p-ERK1/2 decreased quickly after reoxygenation (Figure 4(b)). Total ERK1/2, JNK, and p38 protein levels did not change during H/R exposure. These results indicate that H/R-induced apoptosis in H9c2 cardiomyocytes was accompanied by a quick but transient increase in p-ERK1/2 and sustained/delayed increase in p-JNKs and p-p38 as well as p-AKT. 

### 3.4. RK3 Protects H9c2 Cardiomyocytes from H/R-Induced Cytotoxicity

**Figure 5:**
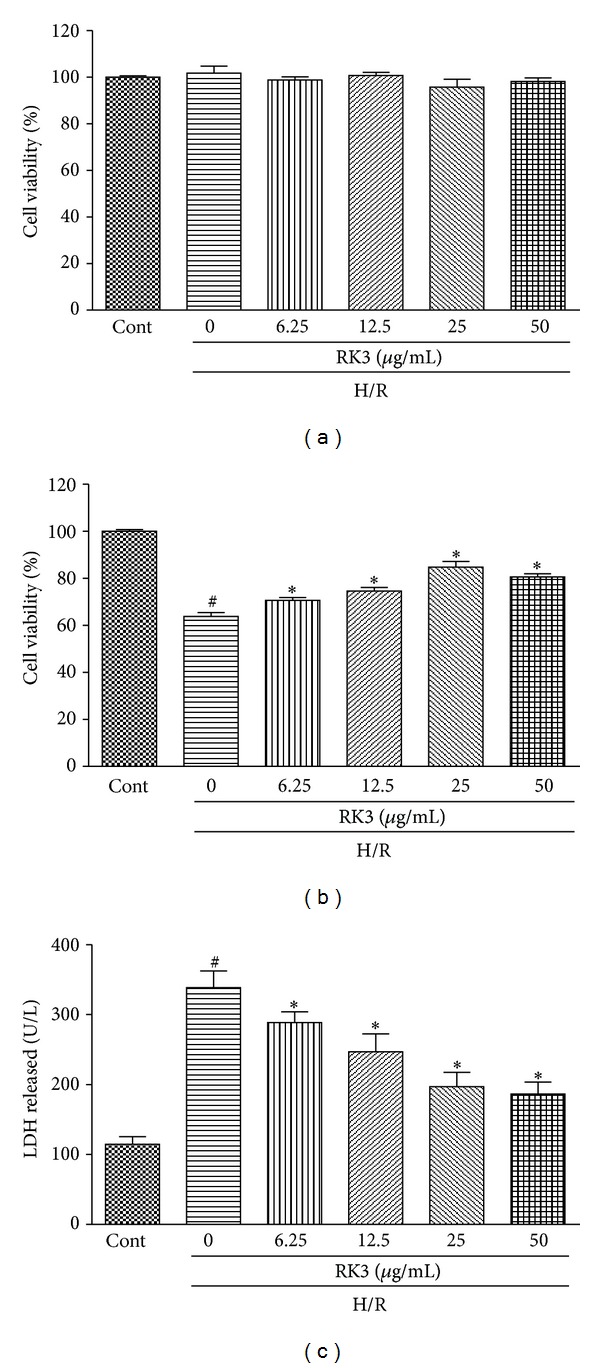
Effects of RK3 on cell viability and LDH release. H9c2 cardiomyocytes were coincubated with different concentrations of RK3 (6.25, 12.5, 25, and 50 *μ*g/mL) for 12 h. Cell viability was determined by CCK8 assay and expressed as a relative percentage of control group (a). Control and RK3-treated cells were further exposed to 6 h of hypoxia followed by 24 h reoxygenation. Cell viability (b) and LDH release (c) were measured. The results are represented as means ± SD from three independent experiments. ^#^ *P* < 0.05 versus control group; *P < 0.05 versus H/R-treated cells.

Before testing the role of RK3 on protection against H/R-induced cell damage, we first analyzed the direct effect of RK3 on H9c2 cardiomyocytes. To this end, cells were treated for 12 h with RK3 in concentrations ranging from 6.25 *μ*g/mL to 50 *μ*g/mL. As shown in Figure 5(a), cell viability revealed no difference between RK3-treated and control groups, demonstrating that none of the tested concentrations of RK3 induced cell injury in H9c2 cardiomyocytes.

H9c2 cardiomyocytes were pretreated with RK3 (6.25 *μ*g/mL to 50 *μ*g/mL) for 12 h then further exposed to H/R. Viability was measured to assess whether treatment with RK3 repressed the cytotoxic effect induced by H/R. The results showed that pretreatment with RK3 (6.25 *μ*g/mL to 25 *μ*g/mL) increased cell viability in a dose-dependent manner. Higher RK3 concentration (up to 50 *μ*g/mL) did not improve cell viability (Figure 5(b)).

In addition, Figure 5(c) showed that pretreatment of cells with RK3 could prevent the appearance of H/R-induced cell death in all the concentrations tested. Treating cells with RK3 (6.25 *μ*g/mL to 25 *μ*g/mL) protected against H/R-induced cell death in a dose-dependent manner. Higher concentration (up to 50 *μ*g/mL) no longer decreased LDH release. Therefore, all subsequent experiments were performed with 25 *μ*g/mL of RK3.

### 3.5. RK3 Protects H9c2 Cardiomyocytes from H/R-Induced Cell Apoptosis

**Figure 6:**
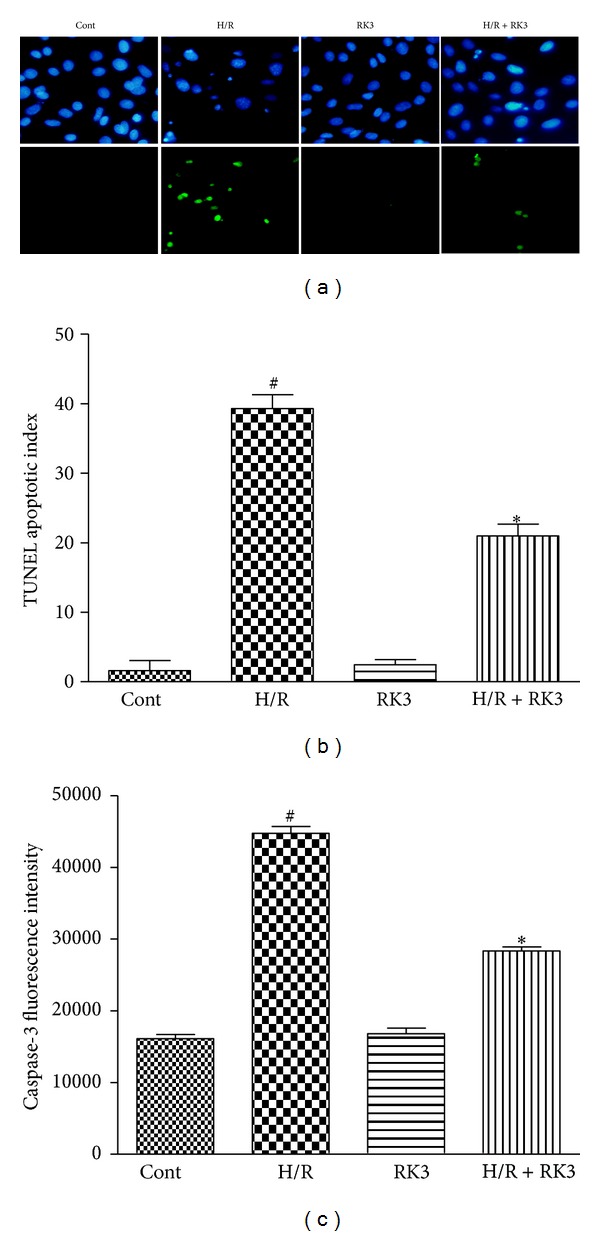
Effects of RK3 on apoptosis induced by H/R. H9c2 cardiomyocytes were pretreated with or without RK3 (25 *μ*g/mL) for 12 h prior to H/R exposure. The internucleosomal DNA fragmentation was determined by TUNEL assay (a). TUNEL apoptotic index was determined by calculating the ratio of TUNEL-positive cells to total cells (b). Caspase-3 activity (units per microgram of protein) was also assayed (c). The results are represented as means ± SD from three independent experiments. ^#^ *P* < 0.05 versus control group; *P < 0.05 versus H/R-treated cells.

TUNEL staining and fluorescence detection of caspase-3 were used in H9c2 cardiomyocytes to confirm whether the protection function of RK3 was connected to apoptosis. As shown in Figure 6(a), H/R-treated H9c2 cardiomyocytes resulted in significant internucleosomal DNA fragmentation. By contrast, pretreatment with RK3 (25 *μ*g/mL) effectively ameliorated the H/R-induced DNA fragmentation. The percentage of TUNEL-positive cells was also calculated, as presented in Figure 6(b). RK3 pretreatment also consistently suppressed the activation of caspase-3 induced by H/R (Figure 6(c)). These results indicate that RK3 can protect H9c2 cardiomyocytes from H/R-induced cell apoptosis.

### 3.6. Effect of RK3 Treatment on H/R-Induced Alterations of Bax and Bcl-2 Proteins in H9c2 Cardiomyocytes

**Figure 7:**
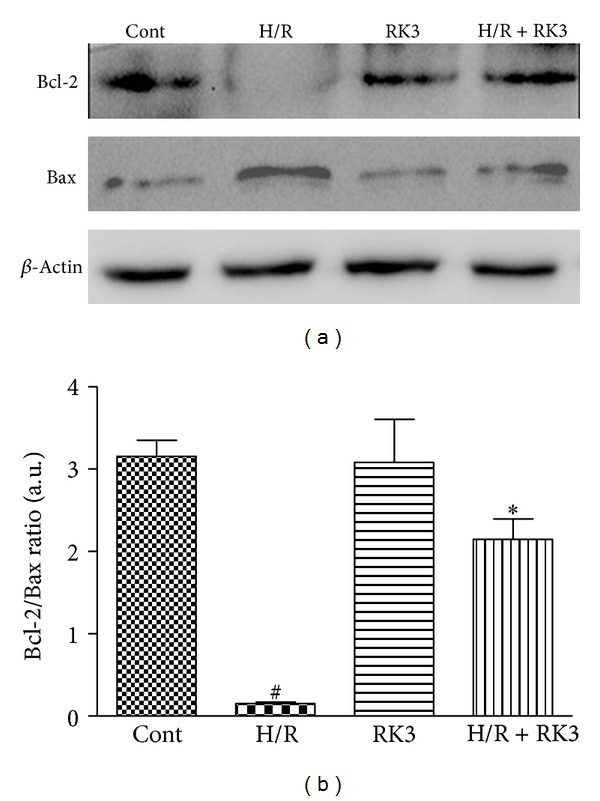
Effects of RK3 and H/R on expressions of Bcl-2 family proteins. The expression levels of Bcl-2 and Bax proteins were detected using an immunoblotting assay (a) and expressed as fold changes over the control (b). The results are represented as mean ± SD from three independent experiments. ^#^ *P* < 0.05 versus control group; *P < 0.05 versus H/R-treated cells.

To investigate whether RK3 could modulate the expression of antiapoptotic Bcl-2 and proapoptotic Bax proteins, Western blot analysis was performed in H9c2 cardiomyocytes exposed to H/R. As shown in Figure 7(a), H/R treatment resulted in a decrease in Bcl-2 protein and an increase in Bax protein compared with control cells.

However, pretreatment with RK3 blocked these changes. Furthermore, the decrease of the Bcl-2-to-Bax ratio in H9c2 cardiomyocytes exposed to H/R was enhanced by RK3 treatment (Figure 7(b)). These findings suggest that RK3 can enhance I/R-induced apoptosis via regulating the expression of Bcl-2 family proteins.

### 3.7. Involvement of AKT Signaling Transduction Pathway and RK3 in H/R-Induced Caspase-3 Activation and Cytotoxicity

**Figure 8:**
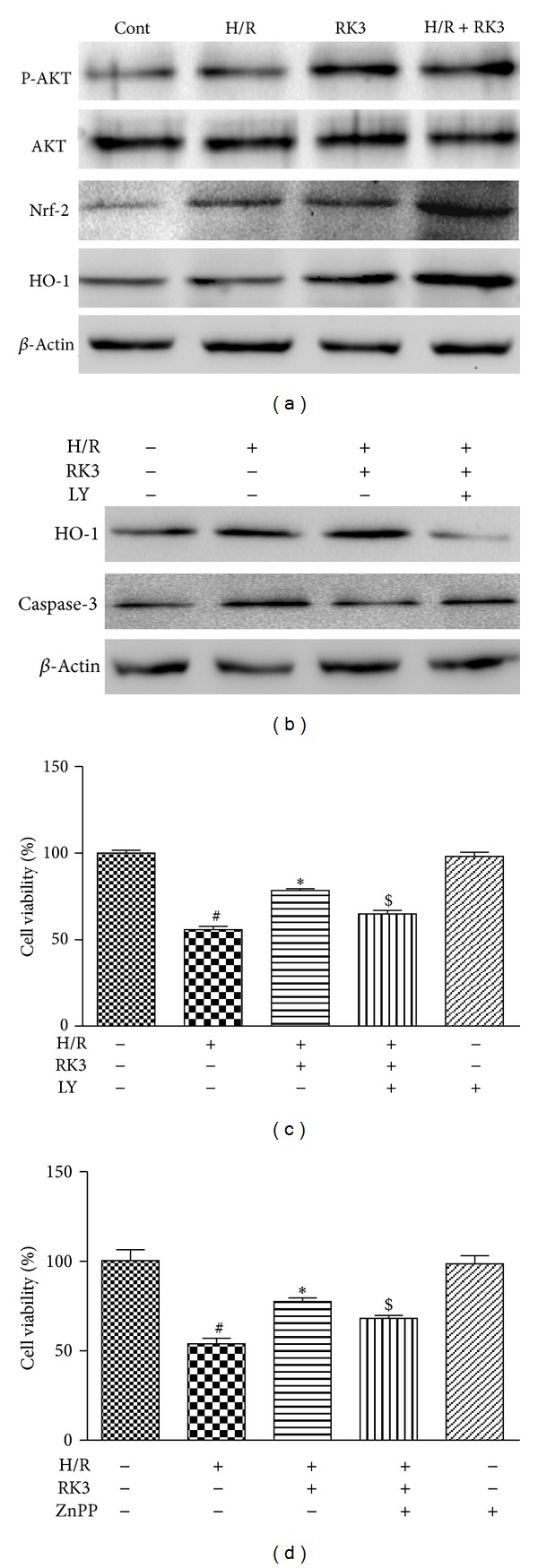
Involvement of AKT signaling transduction pathway and RK3 in H/R-induced caspase-3 activation and cytotoxicity. H9c2 cardiomyocytes were pretreated with or without RK3 (25 *μ*g/mL) for 12 h prior to H/R exposure. The expression levels of ERK1/2, p38, and JNK activity were detected by immunoblotting assay (a). The effects of LY294002 (an AKT-specific inhibitor) on RK3-mediated activation of HO-1, caspase-3, and cell viability in H9c2 cardiomyocytes were measured (b and c). The effect of Znpp (an inhibitor of HO-1 activity) on RK3-mediated cardioprotection was also evaluated by CCK8 assay (d). The results are represented as means ± SD from three independent experiments. ^#^ *P* < 0.05 versus control group; *P < 0.05 versus H/R-treated cells;  ^$^ *P* < 0.05 versus H/R+RK3-treated cells.

The combined PI3K/AKT-Nrf2/HO-1 signaling transduction pathways reportedly play a key role in protecting cells against the toxicity caused by H/R. Thus, we were prompted to evaluate whether RK3 would affect H/R-induced AKT phosphorylation and subsequent Nrf-2 nuclear translocation and HO-1 upregulation in H9c2 cardiomyocytes. As shown in Figure 8(a), RK3 pretreatment increased the protein level of p-AKT significantly, compared with either the control group or the H/R group. Consonantly, H/R slightly upregulated the expressions of HO-1, as well as the accumulation of the nuclear factor Nrf2. Pretreatment of RK3 markedly upregulated the HO-1 and Nrf-2 expressions in cardiomyocytes compared with the control and H/R groups.

The effect of RK3 on the combined PI3K/AKT-Nrf2/HO-1 signaling transduction pathways was further confirmed using a PI3K/AKT inhibitor LY294002 and an inhibitor of HO-1 activity (ZnPP). As shown in Figure 8(b), pretreatment of H9c2 cardiomyocytes with LY294002 significantly increased the expression of caspase-3 and inhibited the upregulation of HO-1 compared with RK3 treatment groups. Furthermore, LY294002 or ZnPP could attenuate the RK3-mediated cardioprotection indicated by decreased cell viability as shown in Figures 8(c) and 8(d). These results suggest that RK3 mediates its cardioprotective effect through the activation of the combined PI3K/AKT/Nrf2/HO-1 signaling transduction pathways in H9c2 cardiomyocytes.

### 3.8. Involvement of ERK1/2, JNK, p38, and RK3 on H/R-Induced Caspase-3 Activation and Cytotoxicity

**Figure 9:**
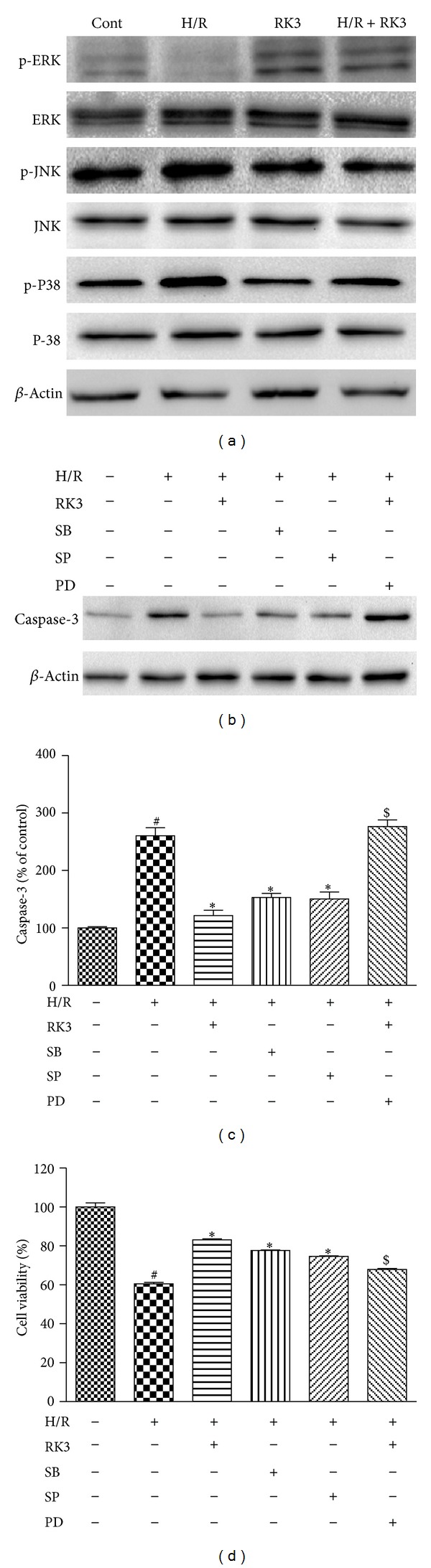
Involvement of ERK1/2, p38, JNK, and RK3 in H/R-induced caspase-3 activation and cytotoxicity. H9c2 cardiomyocytes were pretreated with or without RK3 (25 *μ*g/mL) for 12 h prior to H/R exposure. The expression levels of phosphorylated and total ERK1/2, p38, and JNK were detected by immunoblotting assay (a). The effects of the PD98059 (an ERK1/2-specific inhibitor), SB203580 (a p38-specific inhibitor), and SP600125 (a JNK-specific inhibitor) on caspase-3 activation (b and c) and cell viability (d) were determined. The results are represented as means ± SD from three independent experiments. ^#^ *P* < 0.05 versus control group; *P < 0.05 versus H/R-treated cells;  ^$^ *P* < 0.05 versus H/R+RK3-treated cells.

Accumulated evidence indicates that the MAPK signaling pathways, including the ERK1/2, JNK, and p38 MAPK, play a major role in the apoptosis processes induced by H/R. The expression levels of both total and phosphorylated proteins were evaluated through immunoblotting assay to determine the involvement of the MAPK signaling pathways. As shown in Figure 9(a), RK3 pretreatment increased the protein level of phosphorylated ERK1/2 in H9c2 cardiomyocytes, compared with the H/R group. By contrast, pretreatment with RK3 significantly inhibited the expression of p-JNK and p-p38 induced by H/R.

Furthermore, pretreatment of PD98059 (an ERK-specific inhibitor) attenuated the RK3-mediated cardioprotection. However, pretreatment of SB203580 (a p38-specific inhibitor) or SP98059 (a JNK-specific inhibitor) reduced H/R-induced decline of cell viability and capase-3 activation. (Figures 9(b), 9(c), and 9(d)). All these results indicate that the MAPKs signaling pathway was involved in the antiapoptotic and cytoprotection effect of RK3.

## 4. Discussion

Despite advancements in the pharmacologic and early revascularization therapies over the past two decades, AMI remains a leading cause of death in worldwide epidemics. A major reason for this situation is I/R injury. As with reperfusion, reoxygenation does not completely reverse the hypoxia-induced changes but causes further cardiomyocyte damage [24]. Experimental animal models and pathologic studies in humans show that apoptosis may be responsible for AMI and I/R injury and involved in postinfarction remodeling. In addition, apoptosis is always an important research direction for therapeutic application in AMI since the first description of apoptosis in this pathological process [25–27]. 

RK3 is reportedly present in Radix notoginseng which is generally used as a major ingredient of compound preparation such as Compound Danshen Tablets, used to treat ischemic heart diseases [28]. In this study, we first demonstrated that RK3 can protect H/R-induced H9c2 cardiomyocytes injury. The underlying mechanisms of this protective effect of RK3 are proven to involve AKT and MAPK pathways.

In present study, we first validated that H/R-induced cell damage in H9c2 cardiomyocytes was associated with apoptosis by activation of caspase-3, which is considered a very specific and sensitive apoptotic marker [29]. The morphological changes of apoptotic H9c2 cardiomyocytes induced by H/R were observed by Hoechst 33342/PI staining. In this sense, a time-dependent increase in the percentage of apoptotic cells was detected from 0 h to 24 h of reoxygenation in the model groups. This result suggests that apoptotic events occur in H9c2 cardiomyocytes in response to hypoxia, and they are further evidenced by reoxygenation. The protective effect of RK3 pretreatment is manifested in the increased cell viability and decreased levels of LDH leakage. Moreover, RK3 pretreatment inhibited cell apoptosis, as evidenced by the decrease in caspase-3 activity, TUNEL-positive cells, and Bax expression, as well as the increase in Bcl-2 level. 

AKT and MAPK pathways are reportedly required for cell survival and apoptosis [5, 10]. In this regard, we hypothesized that the cardioprotective effect of RK3 against H/R-induced apoptosis in H9c2 cardiomyocytes is related to a mechanism of AKT and MAPK signaling cascades. As expected, our results showed that RK3 treatment selectively increased the activation of AKT and ERK1/2 and inhibited the activity of JNK and p38. Thus, RK3 may suppress H/R-induced cell apoptosis, at least in part, through modulation of AKT and MAPK signaling pathways.

The PI3K/AKT pathway, a known target of I/R injury, plays a critical role in cell survival [30, 31]. The influence of H/R on the phosphorylation of AKT was first measured in this study. Western blot analysis showed that AKT phosphorylation was notably activated in H9c2 cardiomyocytes exposed to hypoxia and enhanced by reoxygenation. Pretreatment of RK3 increased the protein level of p-AKT significantly, compared with either the control group or the H/R group. Several studies demonstrate that AKT activation can activate Nrf2 and subsequently upregulate the expression of HO-1 [8, 32]. The activated Nrf2 is released from Keap1, which is the inhibitor of Nrf2, and translocates into the nucleus, where it binds to the antioxidant responsive elements (AREs) and accelerates HO-1 expression. HO-1, one of the phase II detoxification enzymes, was reported to play a key role in protecting against I/R induced injury [33]. Western blot results showed an increase in the protein level of Nrf2 in nuclear extracts after H/R. Pretreatment of RK3 enhanced the expression significantly compared with the H/R group. These results demonstrate that RK3 promoted the process of Nrf2 translocation into the nucleus. Accordingly, the expression of HO-1 in the H/R group was slightly upregulated, compared with the control group. Pretreatment of RK3 significantly enhanced the HO-1 upregulation in cardiomyocytes compared with the H/R groups. It is worth noting that treatment of RK3 alone also has a positive effect on Nrf2 translocation and HO-1 upregulation. Furthermore, PI3K/AKT inhibitor LY294002 and HO-1 specific inhibitor ZnPP were used in this study to verify the PI3K/AKT/Nrf2/HO-1 pathway involved in the cardioprotective effect of RK3.

MAPK is a family of serine-threonine protein kinases that is activated in response to various extracellular stimuli, such as I/R injury. Three major subgroups of MAPK are found in mammalian cells: ERK1/2, JNK, and p38, which play key roles in cell growth, differentiation, and apoptosis. Among the MAPKs, JNK and p38 MAPK are preferentially activated in response to a variety of stresses and proapoptotic signals. By contrast, ERK1/2 preferentially responds to stimulation by growth-related signals and is reportedly associated with survival pathways against proapoptotic stimuli [11]. Moreover, several reports demonstrate that JNK and p38 inhibition actually protects cardiac myocytes from I/R-induced apoptosis, whereas the inhibition of ERK1/2 is destructive to cardiomyocytes [10, 34, 35]. However, the role of the MAPK in apoptosis also remains controversial. Some reports indicated that upregulation of p38 activity during postinfarction LV remodeling rescued the failing myocardium. Further investigations showed that postinfarction LV remodeling and heart failure are characterized by a sustained downregulation of p38 kinase activity which is distinctly regulated from other cardiovascular diseases; therefore, we considered the effect of p38 dependents on different diseases and apoptotic stimuli [36].

 Based upon the previous fact, the expressions of p-ERK1/2, p-JNK, and p-p38 were determined at 0, 6, 12, 24, and 48 h after reoxygenation. The results showed that ERK1/2 was activated by 6 h of hypoxia and reduced after reoxygenation. However, JNK and p38 were significantly activated by hypoxia and persistently enhanced during the reoxygenation period. RK3 pretreatment selectively increased the expression of p-ERK1/2 and decreased the upregulation of p-p38 and p-JNK. Moreover, treatment of cells with JNK inhibitor (SP98059) and p38 (SB203580) inhibitor alone significantly decreased the H/R-induced expression of cleaved caspase-3 and the decline of cell viability. Furthermore, combined treatment with RK3 and ERK inhibitor (PD98059) attenuated the antiapoptotic effect mediated by RK3. Overall, these results indicate that the protective effect of RK3 against H/R-induced apoptosis in H9c2 cardiomyocytes partially relies on the inhibition of JNK and p38 and the activation of ERK1/2 signaling pathways.

In this study, the results show that pretreatment with RK3 can prevent H/R-induced apoptosis in H9c2 cardiomyocytes. This cytoprotection is manifested in the increased cell viability and decreased LDH leakage, caspase-3 activity, and TUNEL-positive cells, as well as in the downregulation of Bax expression and upregulation of Bcl-2 protein. The underlying mechanisms of this protective effect of RK3 are connected with AKT and MAPK pathways. 

In conclusion, these findings indicate that RK3 might act as a novel protective agent by inhibiting cardiomyocytes apoptosis in reperfusion therapy for AMI. In addition, RK3 is also a promising agent for the treatment of apoptosis-induced heart injury such as doxorubicine related cardiomyopathy and may have implications for other diseases associated with apoptosis, such as neurodegenerative disorders. 

The limitation of this paper is that these results obtained from in vitro cell experiments needs to be verified in animal models. And the protective effect of RK3 should be confirmed in other apoptosis related disease models for expanding the applied range of RK3 in clinical field. Moreover, pharmacokinetic features of RK3 in animal and human also should be considered.
